# A MIXED METHOD APPROACH TO UNDERSTANDING NURSING STUDENTS' SELF-EFFICACY TO CARE FOR OLDER ADULTS IN GHANA

**DOI:** 10.1093/geroni/igac059.206

**Published:** 2022-12-20

**Authors:** Diana Abudu-Birresborn, Martine Puts, Lynn McCleary, Vida Yakong, Charlene Chu, Lisa Cranley

**Affiliations:** University of Toronto, New York, New York, United States; University of Toronto, Toronto, Ontario, Canada; Brock University, St. Catharines, Ontario, Canada; University for Development Studies, Tamale, Northern, Ghana; University of Toronto, Toronto, Ontario, Canada; University of Toronto, Toronto, Ontario, Canada

## Abstract

To identify students’ motivation, gaps, and opportunities to design effective teaching strategies, it is necessary to examine nursing students' understanding of their self-efficacy to care for persons 60+ years. A mixed-method approach was employed. In phase 1, 170 second-and third-year nursing students from two public Nursing Colleges in Ghana were recruited. A cross-sectional survey included the General Self-efficacy to Care for Older Adults’ scale (GSE-COA), the Kogan’s Attitudes towards Old Peoples scale, and the Knowledge about Older Patients Quiz. Data were analyzed using descriptive statistics in SPSS IBM 26. The results of phase 1, informed the selection of students for phase 2. In phase 2, 17 students were purposefully selected for in-depth semi-interviews. Qualitative data were analyzed using thematic analysis. Both results were then integrated, interpreted, and presented jointly. Students’ mean age was 21yrs (SD=3.73), with 91 (54%) females. Most students 140 (71.0%) had lived with/were currently living with an older adult. The majority 164 (97%), had higher scores for GSE-COA (mean= 107, SD=14.29), indicating high self-efficacy. Qualitative results showed that students’ high self-efficacy was due to their familiarity with older adults at home and their perceived competence in routine nursing care. Students who demonstrated a high sense of self-efficacy were confident and perceived caring for older adults as a duty and responsibility, had experience/exposure to and were comfortable working with older adults. The findings demonstrate an opportunity to design effective teaching strategies to develop and sustain students’ interest and motivation in the care for older adults in Ghana.

